# Integration of Sensors, Controllers and Instruments Using a Novel OPC Architecture

**DOI:** 10.3390/s17071512

**Published:** 2017-06-27

**Authors:** Isaías González, Antonio José Calderón, Antonio Javier Barragán, José Manuel Andújar

**Affiliations:** 1 Department of Electrical Engineering, Electronics and Automation, University of Extremadura, Avenida de Elvas, s/n, 06006 Badajoz, Spain; ajcalde@unex.es; 2 Department of Electronic, Computer Science and Automatic Engineering, University of Huelva, Escuela Técnica Superior, Crta. Huelva-Palos de la Fra, Palos de la Fra, 21919 Huelva, Spain; antonio.barragan@diesia.uhu.es (A.J.B.); andujar@diesia.uhu.es (J.M.A.)

**Keywords:** OPC, sensors, PLC, SCADA, automation, communication, interoperability, smart microgrid, remote laboratory, hardware-in-the-loop

## Abstract

The interconnection between sensors, controllers and instruments through a communication network plays a vital role in the performance and effectiveness of a control system. Since its inception in the 90s, the Object Linking and Embedding for Process Control (OPC) protocol has provided open connectivity for monitoring and automation systems. It has been widely used in several environments such as industrial facilities, building and energy automation, engineering education and many others. This paper presents a novel OPC-based architecture to implement automation systems devoted to R&D and educational activities. The proposal is a novel conceptual framework, structured into four functional layers where the diverse components are categorized aiming to foster the systematic design and implementation of automation systems involving OPC communication. Due to the benefits of OPC, the proposed architecture provides features like open connectivity, reliability, scalability, and flexibility. Furthermore, four successful experimental applications of such an architecture, developed at the University of Extremadura (UEX), are reported. These cases are a proof of concept of the ability of this architecture to support interoperability for different domains. Namely, the automation of energy systems like a smart microgrid and photobioreactor facilities, the implementation of a network-accessible industrial laboratory and the development of an educational hardware-in-the-loop platform are described. All cases include a Programmable Logic Controller (PLC) to automate and control the plant behavior, which exchanges operative data (measurements and signals) with a multiplicity of sensors, instruments and supervisory systems under the structure of the novel OPC architecture. Finally, the main conclusions and open research directions are highlighted.

## 1. Introduction

Supervision, monitoring and automation of technological processes, both for industrial and non-industrial environments, require effective data transmission over communication networks. Such data is related to measuring, acquisition, logging and displaying of information tasks. Moreover, control signals also belong to the exchanged data. By means of digital communications, control units, computers, Human-Machine-Interfaces (HMI), sensors and actuators can be integrated into networks with different topologies in order to share operative data and command signals. Even the increased digital interconnection and computerization of technical systems and components are essential characteristics of technological progress [1]. In fact, in the last decades, great efforts have been done to introduce digital communications in control and field networks [2].

Supervisory systems, called Supervisory Control and Data Acquisition (SCADA) systems, are responsible of controlling and displaying real-time information of the plant behavior and storing the significant measurements and signals for further analysis and interpretation. These systems are traditionally applied in industrial environments but their capabilities make them suitable for any other process that requires monitoring and control functions out of the industry scope [3]. In addition, the developments in Information and Communications Technologies (ICT) have favored the increment of both the data transmission speed and the global use of communication networks [4]. Such advances have given rise to new and important advances in the automation and control domains [5]. Within these networks, there is a paramount requirement, the integration and management of the aforementioned entities (hardware and software) tackling their heterogeneity and interoperability. The latter one, interoperability, is the possibility of system components to interact, and it is a priority for future systems. In the path towards facilitating this functionality, an open and standardized communication platform constitutes a key component [6,7]. In this sense, Object Linking and Embedding for Process Control (OPC) is a technology for standardized data exchange widely used to deal with heterogeneity and interoperability in automation systems. OPC was the name given to the specification of standards developed by an industrial automation industry task force in 1996. It was designed to provide a common communication channel for Personal Computer (PC)-based software applications, mainly SCADA systems, and automation hardware, i.e., a technology for interoperability in process control and manufacturing automation applications. Nowadays, this protocol comprises ten specifications established and managed by the OPC Foundation [8]. The Data Access (DA) specification is the most profusely used and the most recently released, 2006, is the Unified Architecture (UA).

In this context, as a result of ICT and sensors advancements, several challenging concepts for the research community have emerged and are related to communication involved in monitoring and supervision systems. The first concept to consider is the Internet-of-Things (IoT), described as the pervasive and global network, which provides a system for monitoring and control of the physical world [9]. IoT is concerned with the connection of any type of embedded device, which inevitably leads to synergy effects [10]. Within the IoT framework, every device can collect, send and receive data enabled by communication technologies [11]. IoT application is expected to positively impact all aspects of life; however it also entails vulnerabilities due to security and privacy threats [12]. In fact, cyber security research is gaining a lot of focus in last years [5,12,13]. A concern about the IoT adoption is related to the Internet Protocol version 6 (IPv6) that acts as enabling technology to accommodate the huge number of interconnected devices [14]. Precisely the associated costs of IoT devices can inhibit its large-scale implementation, so open source technologies facilitate the deployment of networked sensors and actuators [4] and can contribute to increase the number of devices within the IoT [15].

Data communications can rely on wired or wireless technologies. Wireless communication is gaining attention due to benefits like mobility and flexibility, and will be used to link billions of devices to the IoT [12]. A number of different protocols are available for this data transmission like Bluetooth, ZigBee, Wi-Fi, 3G/4G mobile networks, Z-Wave, Radio Frequency IDentification (RFID), Near Field Communication (NFC), and IPv6 over Low power Wireless Personal Area Networks (6LoWPAN) just to name a few. A challenging issue is to avoid the shortage of spectrum resources that might become a bottleneck for the IoT [16]. Wireless control systems bring advantages in terms of installation complexity, lack of wiring and related costs, enhanced reconfiguration capability of the system, and so on [17]. With sensing goals, Wireless Sensor Networks (WSNs) take advantage of wireless communications to deploy networks of smart sensors, very used in the IoT and CPSs. If control capabilities are added, they are called Wireless Sensor and Actuator Networks (WSANs).

Another new paradigm corresponds to Cloud computing which has virtually unlimited capabilities in terms of storage [18]. The convergence of both technologies, IoT and Cloud, brings important impacts in all fields of science and technology [18]. Information resides in the Cloud and is autonomously accessed and utilized by smart objects connected via the internet [19]. 

Big Data is other intimately linked concept and is referred to large amounts of structured or unstructured data that can be mined and analyzed. Intelligent analytics are supposed to extract meaningful information from the high-volume sets of stored data. These techniques are suitable not only for social and commercial purposes but also for enterprise and industry planning, for instance by means of the Prognostics and Health Management (PHM) framework. IoT devices enable massive collection and supply of information for Big Data analytics.

Regarding industrial applications, their increasing complexity results in a growing amount of data from signal sources that must be acquired, communicated and evaluated. Appropriate supervision imposes to evolve towards distributed intelligent technical systems featured by intelligent sensors and intelligent communications [20]. In this sense, IoT expands the vision of SCADA systems since they are complementary technologies. Even the next generation of SCADA is based on the IoT opportunity, aiming improved functionalities, reduction in costs and easier maintenance taking advantage of Cloud computing. Concerning machine communications, Machine-to-Machine (M2M) systems use different communication technologies to automatically monitor and control remote machines/devices with or without any manual interaction [1]. Much work is being developed to promote connectivity and M2M communications, which is becoming one key topic for the future of industry [21]. In the M2M context, RFID is a well-known ICT technology for identification of objects and people using small cards called tags [22]. RFID helps to monitor data in heterogeneous environments making a tagged object uniquely identifiable; as a consequence RFID is used in many industries with traceability purposes, becoming indispensable in manufacturing and production management [22]. This inexpensive technology can be applied in the IoT ecosystem for vehicle identification, healthcare monitoring, wildlife monitoring and retail logistics [23], favoring the mobility and flexibility needed for IoT proliferation [22]. Even, RFID and NFC are expected to provide a low cost solution to make M2M and IoT communications available for everyone. Diverse works investigate about RFID integration with WSN [24] and with the IoT [16,22,23]. Also, recent examples of joint usage of RFID and OPC are found in [25] and [26] for monitoring assembly systems.

As pointed by Sadok et al. [27], the advent of advances in IoT and M2M communications has paved the way for the convergence of the two worlds—Internet and industrial systems. Due to such arising convergence, there is a great deal of work to develop new standard architectures for industrial networks and middleware [27]. In fact, Industrial Internet-of-Things (IIoT) is the term used to refer to the IoT application in the industrial context and implies the use of sensors and actuators, control systems, M2M communications, data analytics, and security mechanisms [28]. Thanks to these developments, IoT will be one of the main sources of the so called Industrial Big Data [28], and Cloud will enable to store it for long time and to perform complex analyses on it [18]. OPC standard is considered for safe and secure communication for IoT devices [29], to empower the interfacing with existing information technology tools in IIoT environments [28], and as information carrier for IoT and next generation industrial communications [30].

Under this perspective, another challenging concept emerges, the Cyber-Physical Systems (CPS) which implies that the machines have advanced communication and intelligent capabilities. In CPS embedded devices are connected through the network to sense, monitor and actuate physical elements in the real world [31]. As a consequence, in the era of CPS dominated industrial infrastructures, a huge volume of data is collected in real time by a vast number of networked sensors that need to be analyzed in real time [32]. The production systems of the future are envisioned to be developed as Cyber-Physical Production Systems (CPPS), which result from the integration of the IoT and the CPS concepts. In fact, OPC provides a reliable, robust and high performance communication means in the domain of CPS [33] where it is a major contender for the application protocol [34]. As a consequence, it is expected to play an important role in the CPPS development as a language for standardization of communications in a M2M context [35], even connecting and transmitting information between different CPPS [36].

Furthermore, the IoT is expected to bring about a fourth industrial revolution under the flagship of the IIoT [37], commonly referred with the term Industrie 4.0 resulting from an initiative of the German government [38]. In other words, it is envisioned that IoT and CPPS can bring a similar big jump as those caused by the mechanical loom (first industrial revolution), by the Ford assembly line (second revolution), and the first programmable logic controller (third revolution) [31]. In this sphere, the advantages of OPC are able to address the challenges introduced by the Industrie 4.0 [39], being identified as a key technology to handle heterogeneous machine interoperability [21] and to achieve vertical integration in highly modular and multi-vendor production line [40]. 

An example of emerging CPS is constituted by the Smart Grids (SGs), the intelligent power grids are a result of a co-evolution of energy systems and ICT [41]. Apart from generation and distribution of electric power, SGs are able to store, communicate and make decisions [42]. The integration of advanced technologies in communication, smart energy metering and intelligent control confers such capabilities to SGs [43]. Even more, the term Internet of Energy arises from the convergence of the visions of IoT and SG [44]. For energy automation, OPC is gaining importance as an automation standard in the field of the future power systems [45], contributing to develop new types of intelligent systems integrations in SGs [46], where it is signaled as one of the core standards [13].

In recent years, there has been a shift from systems based around the interconnection of physical components where transmitted information has been used with control purposes, towards systems in which information constitutes the heart of the system [19]. Several expectations towards the future technological scenario are handled: ubiquitous connectivity, local intelligence, safety, self-organization, flexibility, massive data monitoring, and efficiency, just to name a few. All of the mentioned recent and future trends rely on a common feature: effective and reliable communication. Furthermore, to achieve such core tasks, the heterogeneity and interoperability of the involved entities (hardware and software) must be addressed.

A big challenge in IoT and Cloud scopes is related to the wide heterogeneity of devices, operating systems, platforms, and services available and possibly used for new or improved applications [18]. The lack of standards is actually considered as a big issue, so research efforts must be performed in the direction of defining standard protocols, languages, and methodologies to enable the full potential of such concepts [18]. In the same sense, SGs are heterogeneous networks, so the existing utility proprietary protocols and evolving communication protocols must converge into a common protocol platform [47]. The IoT and Cloud paradigms are supposed to solve many interoperability issues, but it will not be achieved automatically so there is a need to know the current state [25]. OPC is already playing an important role in the development and establishment of such standardized and open solutions for proper interoperability management.

Deep analysis of the aforementioned technological trends is beyond the scope goal of this paper, but the relevance of OPC for a proper interoperability management in such advanced systems has been clearly stated. 

Based on client/server architecture, OPC aims at reducing dependencies between systems, providing a connectivity layer mainly devoted to vertical data integration in the automation domain. When a manufacturer provides an OPC server for its devices, these can be accessed by any OPC client software. i.e., this standard is the channel to communicate the higher level software applications with the underlying field devices. Interfacing with physical devices that support OPC technology provides connectivity with all kind of industrial and instrumentation apparatus overcoming proprietary limits. The OPC servers translate the information of one device to a unique language; therefore other devices or software programs have access to that information via their own OPC interfaces [48].

Within the physical devices, Programmable Logic Controllers (PLCs) are the traditionally used automation units due to their reliable and robust operation. They are real-time industrial electronic devices hardened for factory environments. Apart from industrial facilities, PLCs are used in building automation [49], Renewable Energy Sources (RES) systems and SGs [50,51], and so on [52]. The PLC’s ability to support a range of communication methods makes it an ideal control and data acquisition device for a wide variety of applications. Apart from PLCs, other field devices manage data which can be interfaced through OPC like Data Acquisition Cards (DAQs), Remote Terminal Units (RTUs), robot controllers, gateways, and other industrial apparatus for measurement, instrumentation, data transmission and/or control.

PLCs are usually integrated by means of fieldbuses, which use industrial communication protocols to interconnect controllers, HMIs, SCADA systems, sensors and instruments into a network of different topologies. Examples of widespread applied fieldbuses are PROcess FIeld BUS (PROFIBUS), PROcess FIeld NETwork (PROFINET), Actuator Sensor Interface (AS-i), Fieldbus Foundation, Modbus, Highway Addressable Remote Transducer (HART), Controller Area Network (CAN), Ethernet for Control Automation (EtherCAT) or Industrial Ethernet.

Concerning supervisory control tasks, in a vertical integration approach, the SCADA/monitoring system maps the field devices and accesses to their memory positions via OPC. Horizontal integration is also achieved since both software applications and hardware devices can share information inside the same layer. For instance, information can be shared between PLCs and other field devices from different manufacturers. Whether it is for vertical or horizontal data flow, OPC interface makes system integration in a heterogeneous environment simpler, as an OPC client can connect to numerous OPC servers.

In R&D and educational activities there is a need for implementing automation systems that share common features like reliability, scalability, flexibility and open connectivity. To solve this issue, a novel OPC-based architecture for automation systems is proposed in this paper. It uses the OPC abstraction layer as a means to effectively communicate data between field devices and supervisory systems in a vertical integration scheme. Such an architecture has been developed in order to implement efficient automation systems applied in different scopes. 

In the University of Extremadura (henceforth referred to as UEX) during last years the OPC technology has been used as fundamental element to develop several demonstrative systems. The utilization of OPC interface was motivated by the need of using PLCs of the manufacturer Siemens jointly with a SCADA system developed in LabVIEW for R&D projects. Thanks to the capabilities of the OPC, their integration was easy and effective. A set of these experimental application cases is exposed as recent examples of the suitability of the proposed architecture. To this aim, application cases related to automation of energy systems like a smart microgrid and a photobioreactor plants, implementation of an industrial networked remote laboratory and development of an educational hardware-in-the-loop platform are reported. Such systems show the OPC standard as an effective medium of integration of networks of controllers, sensor and instruments, regardless of the particular nature of the automated/supervised process. 

In this sense, the contribution of this paper is twofold. On the one hand, a novel OPC-based architecture for automation systems is presented. On the other hand, a set of experimental applications validate the proposed architecture. The target group of the paper is engineers and researchers, mainly in automation- and monitoring-related tasks, who may find such a paper as a valuable resource regardless of the specific application domain. Indeed, OPC standard is an integrated part of academic and research tools such as LabVIEW and Matlab. 

The main benefits of the OPC-based architecture are listed as follows:

- Communication protocol supported by most of modern and even legacy monitoring and automation hardware and software.
- There is no inherent limitation on the number of OPC client/server connections.
- Ability to accommodate remote sensors and instruments through industrial field buses.
- Abstraction of field devices and proprietary drivers, providing open connectivity with instrumentation and industrial hardware.
- Generality, scalability and modularity derived from this abstraction.
- Short development and deployment time due to easy configuration.
- Reliability and stability proved in several applications.
- Improvements in specifications and capabilities.

The remainder of the paper is organized as follows: after this Introduction, Section 2 deals with the materials and methods used for the implementation of the reported systems. Section 3 presents a novel architecture to develop OPC-based systems regardless of the specific scope. In Section 4, four OPC-based experimental applications developed in the UEX are described as a proof of concept of the proposed architecture. This section reflects the depth of the work done both hardware and software to manage the diverse information flows. Finally, main conclusions and further works are addressed in Section 5.

## 2. Materials and Methods

The systems described in Section 4 constitute examples of OPC-enabled vertical integration. In the present section, the hardware and software elements used for their implementation are described. All of the exposed systems include a Siemens PLC, software packages to create the OPC server and the OPC client, and a PC for configuration tasks. This PC, named as Data Exchange and Configuration Laboratory Server (DECLS), is placed in the laboratory or control room where the user/operator accesses to supervisory applications The OPC DA specification is the one chosen for these applications since it resolves effectively the communication and interoperability issues. The OPC server component provides access to the most current value for a given data point via groups of related items where each item has a set of properties [53].

With regard to the signals management, connected to the digital and analog inputs and outputs ports of the PLCs, numerous sensors and instruments are exploited to acquire and apply, respectively, the signals involved in the automated operation of the plants. The required data gathering is performed by SCADA systems linked with such PLCs. Besides, the PLCs include Transmission Control Protocol/Internet Protocol (TCP/IP) connectivity so the devices are connected to the Local Area Network (LAN) in order to share operative data. Table 1 summarizes the exposed cases, indicating the application scope, the OPC role, the software involved for both client and server, and the hardware appliances. 

This section is divided into four subsections, each one devoted to describe the hardware and software elements of the reported experimental cases

### 2.1. Smart Microgrid

In the context of SGs, nowadays severe efforts are being carried out towards development of technologies related with SG basic elements: energy generation and distribution, energy management, automation and monitoring, value-added services, information and communication infrastructure, and participatory dimension [41]. SGs have the strong requisite of an efficient communication infrastructure to monitor and control the distributed energy resources states (current, voltage and power) [54]. Despite of the definition of communication standards focused on electric utilities like the IEC 61850, IEC 61970 or Distributed Network Protocol 3 (DNP3), OPC is profusely used for SGs and noticeably for microgrids as demonstrated by the diverse recent works in such sense. Microgrids can be defined as small scale SGs which can be autonomous or grid-tied [55]. They are supposed to play a key role in the evolution of SGs, becoming prototypes for SGs sites of the future [56]. The operation of islanded microgrid can effectively mitigate the detrimental situations raised by the utility grid, reducing the operation cost and enhancing the reliability when a power shortage occurs [57]. Microgrids integrate both physical elements in the power grid and cyber elements (sensor networks, communication networks, and computation core) to make the power grid operation effective [57]. In this regard, various works deal with microgrids automation using OPC [58,59,60,61].

**Figure 1:**
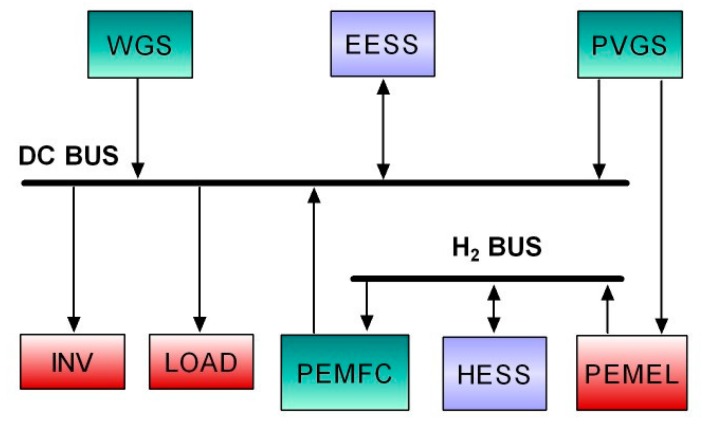
Block diagram of the experimental smart microgrid.

**Figure 2:**
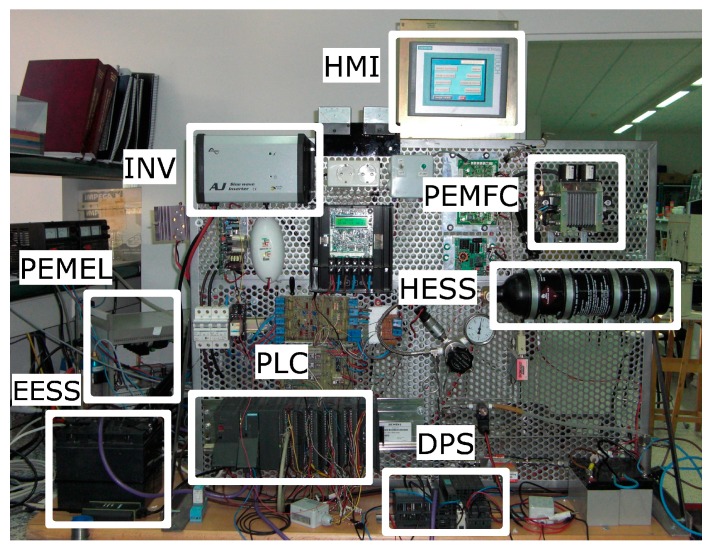
Experimental setup of the smart microgrid.

An experimental smart microgrid is the first OPC-based application case. This microgrid combines RES and hydrogen in order to achieve a self-sufficient isolated and zero-carbon operation. Figure 1 depicts the block diagram of the smart microgrid and Figure 2 shows a snapshot of the experimental laboratory setup. The RES encompasses a Photovoltaic Generator System (PVGS), composed by a set of mono-crystalline photovoltaic modules, and a Wind Generator System (WGS). Both generators are connected to a gelled lead-acid battery which acts as Electrochemical Energy Storage System (EESS). This system constitutes a DC voltage bus that hosts the electrical flows. A DC load (LOAD) and an inverter (INV) are supplied by the generators. On the other hand, the hydrogen flows are hosted by the hydrogen bus, the called Hydrogen Energy Storage System (HESS), i.e., a metal-hydride tank for hydrogen storage. Two devices are devoted to produce and consume hydrogen, i.e., a Polymer Electrolyte Membrane (PEM) Electrolyzer (PEMEL) and a PEM Fuel Cell (PEMFC). Refer to [62] for further details.

A set of sensors measure irradiation, wind speed, current, voltage, hydrogen flow, pressure and temperature are connected to a supervisory system and provide the information required to manage the electrical and energetic interactions. Such supervisory system comprises a Siemens S7-300 PLC [63] and an HMI. The interactions between the microgrid components and the energy flows between them are coordinated and supervised by such system in order to achieve a stable operation with high performance. In this sense, the main goal is to supply the load maximizing the utilization of the RES, so the PEMEL uses the surplus of solar energy to produce hydrogen. Consequently, the PEMEL management plays a key role in the performance of the microgrid [64,65]. Table 2 lists the magnitudes involved in the system and the corresponding devices.

A Fuzzy Logic-Based Controller (FLBC) has been integrated in the control structure. The FLBC is responsible of establishing in real time the operating point of the PEMEL according to the existing technological and meteorological conditions. The PEMEL is fed by the PVGS through a DC/DC converter. The FLBC output acts over this last device, adapting the hydrogen production to the available energy.

The software packages involved in this application case are the well-known Matlab/Simulink and Siemens WinCC flexible [66]. The FLBC is implemented using Simulink whereas a WinCC flexible Runtime program retrieves the operational data. Regarding the field level, most of the sensors are directly wired to the PLC whereas the rest of them are connected by means of a Decentralized Periphery Station (DPS) Siemens ET 200S.

### 2.2. Biomass Photobioreactor

**Figure 3:**
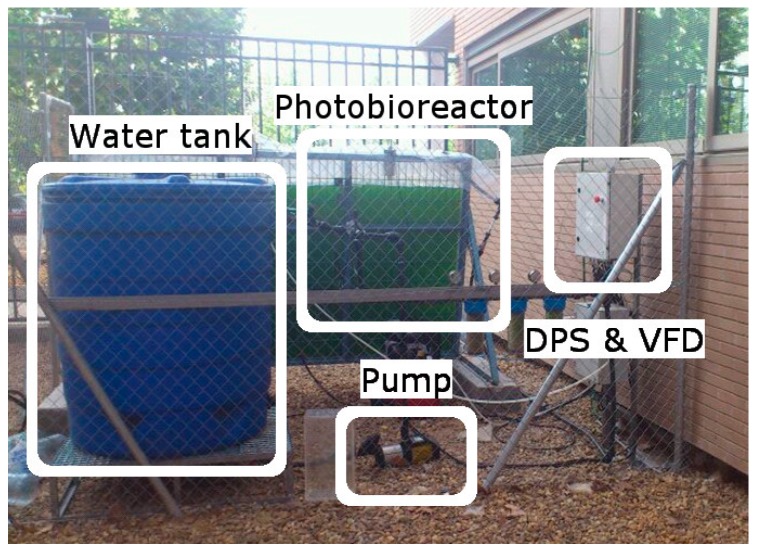
Experimental setup of the automated photobioreactor.

The second case consists on the automation and supervision of a plate photobioreactor for biomass production. This kind of RES is receiving increasing attention and can be produced from microalgae, which has high productivity potential, less competition with food production and less negative impact on the environment when compared with other biomass feedstock options [67,68]. A previous example of using OPC for controlling biomass production in bioreactors is found in [69]. Figure 3 shows the experimental setup of the developed photobioreactor devoted to microalgae culture for biomass production.

The supervisory system comprises a PC-based SCADA platform, a Siemens S7-1200 PLC [70] and a DPS ET 200S. The software package Laboratory Virtual Instrumentation Engineering Workbench (LabVIEW) of National Instruments (NI) [71] has been chosen to develop the SCADA due to the powerful built-in functions and the graphical programing language that provides. The OPC server has been created with the NI OPC Servers package, and the Datalogging and Supervisory Control (DSC) module of LabVIEW has been required during the design of the SCADA to carry out the OPC link.

**Figure 4:**
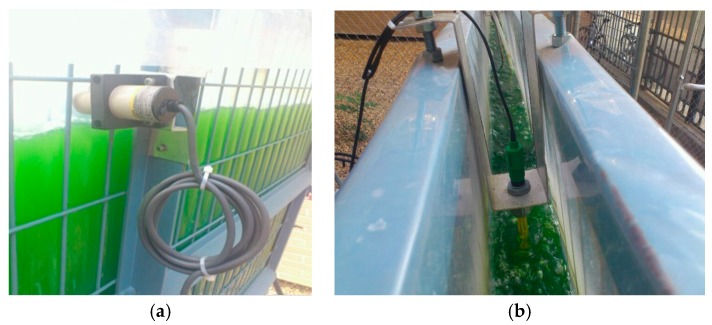
Detail of sensors in the experimental setup: (**a**) Placement of level sensor; (**b**) Placement of pH sensor.

An additional device is a touch operator panel that implements the HMI function placed in the facility location. A set of sensors and actuators are required, namely temperature and pH sensors, water level detector, pumps and electro valves. The main parameters to be controlled in the growing process of microalgae are CO_2_ level and temperature. Figure 4 shows the detail of the placement of the level sensor (Figure 4a) and the pH sensor (Figure 4b). Both sensors and actuators are connected through a DPS, and pumps for the filling and harvesting processes are driven by means of a Variable Frequency Drive (VFD). 

### 2.3. Networked Remote Laboratory

A Remote Laboratory (RL) can be defined as an environment whose function is to control a physical system remotely, aiming to teleoperate a real system, to perform experiments and to access measurement data over the network [72]. The development of RLs has received a great deal of attention in recent years both for R&D activities and education. In-depth analyses of RLs application for science, engineering and control education are addressed in [73]. Some interesting examples of RLs built around the OPC link are reported in [74,75,76,77,78,79]. 

**Figure 5:**
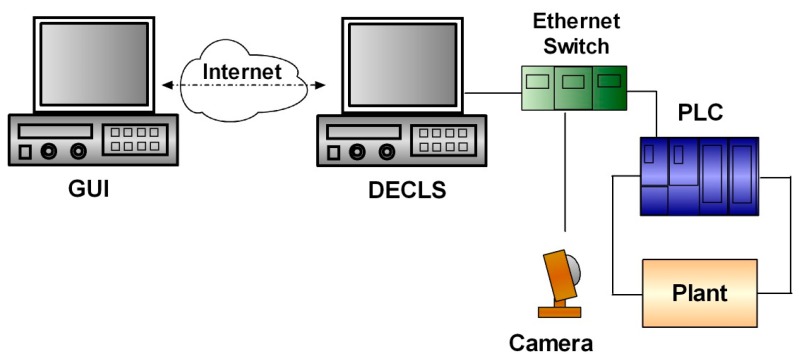
Block diagram of the NRL architecture.

A Networked RL (NRL) has been developed integrating a set of industrial apparatus and interfaces to conduct remote experiments in the field of control, automation and supervision. Figure 5 shows a block diagram of the developed NRL. The DECLS carries out the OPC-based communication tasks between the remote GUI and the local controller, a PLC. Such PLC governs a physical plant, and a camera provides video and audio feedback. These three components are integrated in the LAN of the Industrial Engineering School (IES) by means of an Ethernet switch. Refer to [80] for further details.

**Figure 6:**
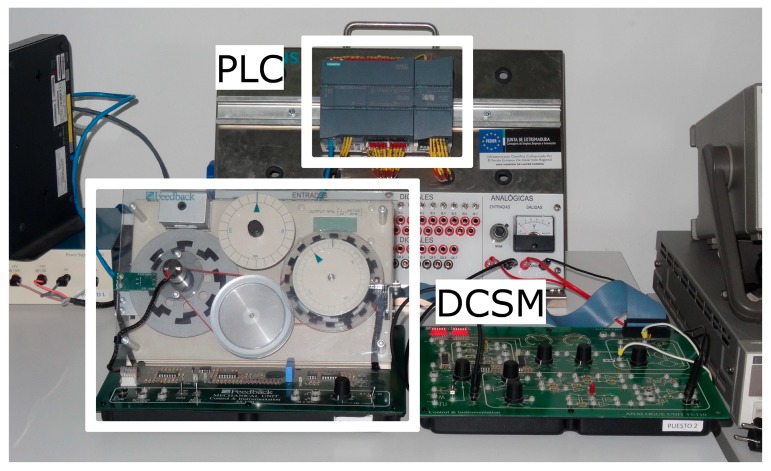
Experimental setup of the physical plant automated by the PLC and accessible through the NRL.

Specifically, a Siemens S7-1200 PLC acts over a plant which consists on a DC servomotor (DCSM). Both control and data acquisition are performed by the PLC so the DCSM is directly wired to it. The implemented algorithm is a FLBC, so that this controller provides a voltage command signal to the DCSM through an analogue output module to control its speed. The snapshot of the experimental setup is shown in Figure 6.

In the remote user side, the GUI has been developed with the open-source authoring tool software Easy Java Simulation (EJS) [81]. This software is devoted to design discrete simulations for supporting interactive virtual and remote laboratories. The GUI includes the elements required to perform both the numerical and visual tracking of the plant behavior, and the online adjustment of the controller parameters. The consequent effects on the DCSM operation are also observed in real-time. The GUI exchanges information with a LabVIEW Virtual Instrument (VI).

The DSC module and the NI OPC Servers are needed to establish the OPC connection. As mentioned, a PC plays the role of DECLS hosting the NI OPC server, the LabVIEW VI, and the software for configuring the PLC.

### 2.4. Hardware-In-the–Loop Platform

A powerful combination of physical controllers and simulated plants can be achieved using OPC as communication channel under the configuration called Hardware-In-the-Loop (HIL). This technique consists on applying a physical controller to a simulation of the plant in real-time. The controller is real (PLC, microcontroller, etc.) whereas the plant is virtual, modelled by a software. The controller behaviour is checked since it ignores that the process is not real. The main utility of this technique relies on the tuning of the controller parameters off-line. Also, disturbances or failures of the process can be simulated to improve the reliability and robustness of the controller. This configuration reduces costs because there is no need of physical equipment to check the controller. Taking advantage of the HIL possibilities, OPC has been used as communication means within such a framework in various works [82,83,84]. 

**Figure 7:**
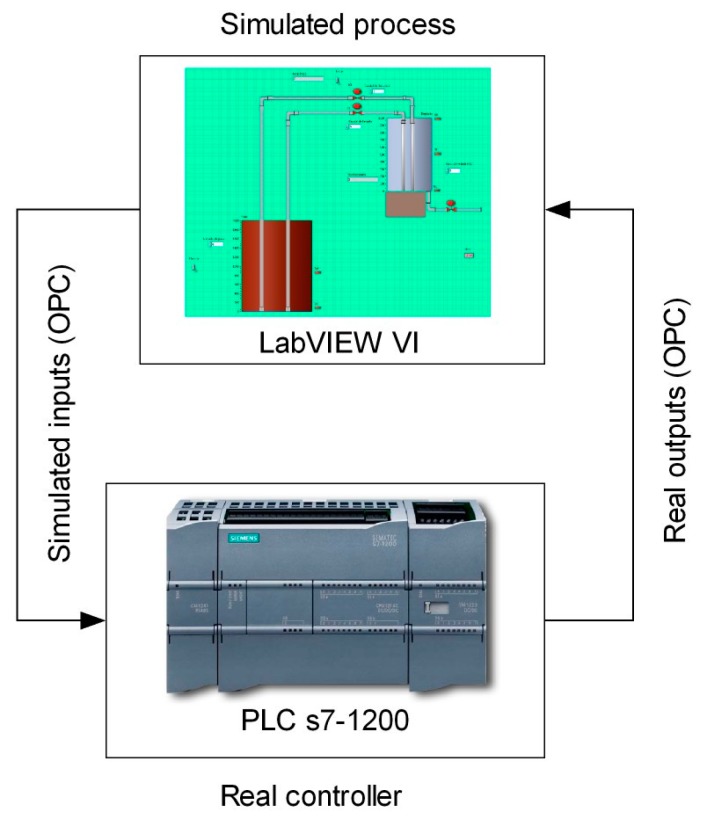
Graphical scheme of the HIL platform components.

In the present work, the fourth experimental case is an OPC-based HIL platform developed with educational purposes. At hardware level, a Siemens S7-1200 PLC is used, whereas LabVIEW is the software responsible of generating a simulation. Data exchange has been configured using the NI DSC module and the NI OPC Servers package. Figure 7 summarizes graphically the interconnected elements to constitute such platform. 

## 3. Proposed Architecture

A novel OPC-based hardware-software architecture is proposed to establish an effective communication and tackle the interoperability of the entities involved in automation systems. Such an architecture enables a seamless integration of sensors, controllers and instruments through the OPC standard. 

A brief background about this kind of proposals in the scientific literature is given below. A number of conceptual architectures/frameworks are proposed for automation purposes, based on existent protocols, to accommodate the emerging trends. For instance, Espí-Beltrán et al. [2] propose a model based on the Service-Oriented-Architecture (SOA) for industrial applications evaluating the HyperText Transfer Protocol (HTTP) and the Constrained Application Protocol (CoAP); an architecture for vertical integration in the context of IIoT is developed in [37]; An IoT-enabled architecture is designed by Ziogou et al. [85] to transform the traditional industrial automation infrastructure. Regarding the use of OPC, in [86] a SOA based on OPC is proposed for industrial automation; Toro et al. [35] present a modular architecture devoted to CPPS and Industry 4.0 approaches where OPC is used for data communication. These architectures share the common feature of being divided into levels or layers (including physical and software elements) interconnected by means of communication channels and protocols. In addition, all of those works emphasize the crucial role of standardized and open communication protocols.

**Figure 8:**
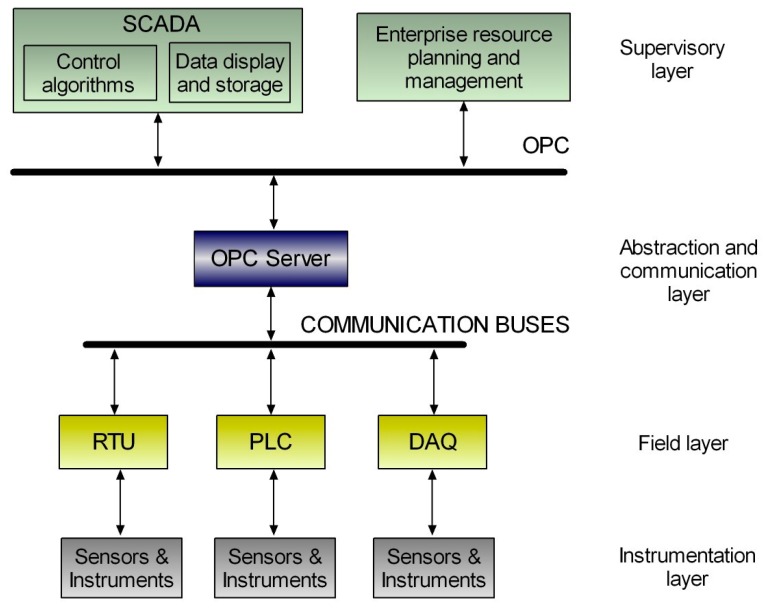
Topology of the proposed architecture to integrate sensors, controllers and instruments through OPC communication in a vertical integration scheme.

Inspired in the automation pyramid, the proposed architecture comprises four layers or levels. Figure 8 illustrates this four tier topology deployed for vertical integration where OPC utilization allows configuring a framework which abstracts the multipurpose client applications from the underlying automation and instrumentation technologies. The top layer plays, as OPC client, a supervisory role that covers process supervision and planning software applications such as SCADA systems, Human-Machine Interfaces (HMI), Graphical User Interfaces (GUI) and maintenance and enterprise resource management applications. On the other hand, the physical resource layer, or field layer, is composed by the hardware equipment, namely PLCs, and so on. The field devices play the role of data sources while the software entities act as data sinks. The lowest level is the instrumentation layer, which includes sensors, actuators and other instruments devoted to measure the process magnitudes required for control and monitoring purposes as well as to apply the control commands for a proper automated operation.

The OPC interface materializes the middleware, i.e., an abstraction and communication layer to perform data exchange between the supervisory and the field layers. An OPC server makes the variables of the field devices available to the OPC clients. Hence, the client applications access and manage the field information without need of knowledge about the physical nature of data sources. A bidirectional flow of information is established so read and write operations are performed through the OPC link. The OPC server runs in the DECLS, so this equipment belongs to the abstraction and communication layer. Furthermore, a common additional sub layer consists of communication buses, which are considered within the abstraction and communication layer.

In the presented architecture, the data logging and storage tasks are carried out by the SCADA system. A database is generated and fed by such SCADA, accumulating the acquired and exchanged data for further treatment. Likewise, online remote access for distant monitoring/supervision through the network is achieved using the remote connection options of the SCADA system via web browser or native desktop interfaces. 

The field layer covers the functions of data acquisition and control of the process behavior. The core device in this level is the PLC due to its widespread utilization and reliable operation. However, instead of a single controller/PLC, numerous and different controllers can be easily integrated by means of communication buses as a network of controllers. As exposed, other physical equipment like RTUs, robot controllers, DAQs, etc., can act as data sources. 

Concerning sensors and instruments, they are connected directly to the controllers or to the RTUs. Smart sensor networks can also be integrated via OPC as part of the instrumentation layer. 

The upper and lower layers can be deeply modified independently without affecting each other. The corresponding modifications are performed in the abstraction and communication layer, facilitating the variations and improvements in existing facilities.

The framework satisfies the target of most of automation and supervision systems. Both, a simple case of only monitoring and a more complex case of advanced control algorithms and sophisticated data treatment, can be carried out using the exchanged data via OPC. Regardless of the complexity of the tasks to develop, they are supported by OPC communication. 

The novelty of the proposed OPC-based architecture relies in the division of the different elements into four layers or categories, aiming to foster the systematic design and implementation of automation systems involving OPC communication. In other words, our proposal goes beyond simply applying a communication protocol, a conceptual framework is presented, structured into functional layers where the diverse components are categorized. It offers advantages in the design, implementation, maintenance and expansion of the sensing and automation infrastructure.

In many cases, the handling of interoperability is complex and involves specialized skills related to programming and/or communications, making difficult to build an efficient solution. The presented proposal is intended to be applied by automation and supervision researchers, offering the advantage of affordable and easy configuration without deep expertise.

The described layers can also be subdivided to cover specific needs. For instance, the top layer can accommodate a different topology if required, SCADA applications can be hierarchically placed bellow the maintenance and enterprise resource management applications. The data flow with the field layer would take place through the abstraction and communication layer equally, so the proposal would still remain valid in this case.

It should be noted that this approach is intended to be valid for the OPC UA specification and future newer releases. In the same sense, the architecture can be expanded by integrating other communication protocols in the abstraction layer, enhancing the connectivity for components (hard and soft) that do not support OPC. Even, the architecture is not limited to centralized schemes due to the fact that the abstraction and communication layer can host more than one OPC server, enabling decentralized approaches aligned with the IIoT and CPPS paradigms. 

Indeed, the OPC server can be cloud-hosted, enabling advantages like ubiquitous access from the network and easy accommodation of IoT devices. In this sense, the architecture is able to integrate IoT-based and RFID sensors, that would be located in the instrumentation layer and share information with the higher layers through OPC. Recent examples of integration of OPC with RFID and IoT technologies can be found in [25,26,28,29,30].

Many powerful architectures are designed and simulated or even experimentally tested in prototypes; however, their practical large-scale implementation is limited due to immature state of development, complexity and/or economic reasons. On the contrary, the proposed architecture has been experimentally validated with real components and magnitudes, with easy configuration, as described in the next section.

To demonstrate the suitability and features of the presented architecture, four experimental applications have been developed for different scopes and using diverse tools.

## 4. Experimentation

In this section a set of experimental systems integrating controllers, sensor networks and instruments by means of the proposed architecture, are exposed. For every case, the communication network is described, emphasizing the role of the OPC interface and the exchanged signals. As aforementioned, these application cases have been developed to solve diverse R&D and educational activities as well as to demonstrate the suitability and features of the presented architecture.

### 4.1. Management of Smart Microgrid

In this scheme, to solve the data exchange, the OPC protocol performs the interfacing between the FLBC and the PLC. The command signal generated by the FLBC is written in the PLC memory via OPC and is physically applied to the PEMEL through a voltage analogue output of the PLC. The signals exchanged via OPC are listed in Table 3.

**Figure 9:**
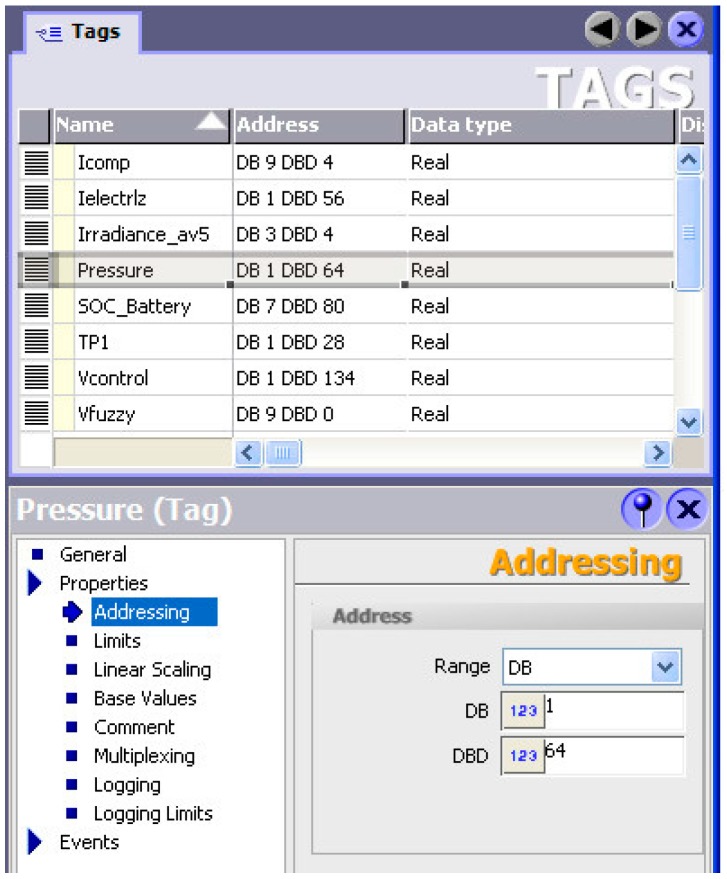
Configuration of tags in the OPC server provided by WinCC flexible.

The WinCC flexible program implements the OPC server to share information between the FLBC and the PLC. Such OPC server accesses to the PLC data blocks where the measurements are stored. The OPC client has such information available to process it. In this case, the supervisory application is the FLBC, i.e., it is the OPC client. The described software applications are continuously running in the DECLS. A group of tags has been created to accommodate the exchanged signals (see Figure 9).

**Figure 10:**
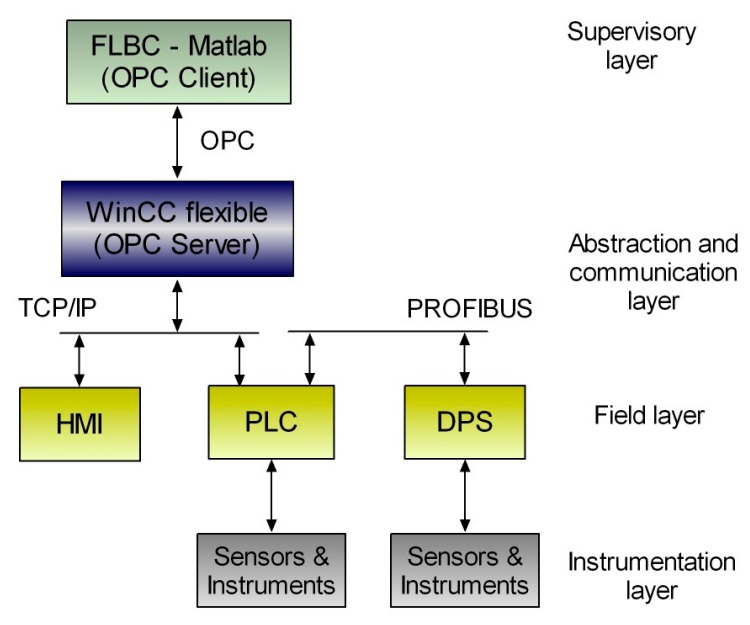
OPC-based communication network for the microgrid.

The PLC, the HMI and the DECLS are linked via Ethernet TCP/IP. The HMI implements the function of monitoring system, displaying continuously real-time data and performing the data logging of the diverse signals for further analysis. The DPS is connected to the PLC by means of the field bus PROFIBUS. Figure 10 portrays the developed communication network according to the proposed architecture. 

### 4.2. Automation of a Biomass Photobioreactor

In this application, the OPC communication channel supports the information flow between the SCADA system and the PLC. The most relevant signal to be measured is the pH, as the CO_2_ injection depends on its value. Hence, the management strategy is intended to perform the feeding of CO_2_ and the corresponding filling and harvesting cycles of the photobioreactor. To this aim, from the measurements provided by the sensors, the SCADA system determines the switching conditions of the valves that introduce CO_2_ in the photobioreactor according to a control algorithm. In addition, the user is able to use a manual mode in order to modify commands through the SCADA, manage the system behavior for maintenance tasks or different trials. Table 4 shows the magnitudes involved in this system and the corresponding devices.

**Figure 11:**
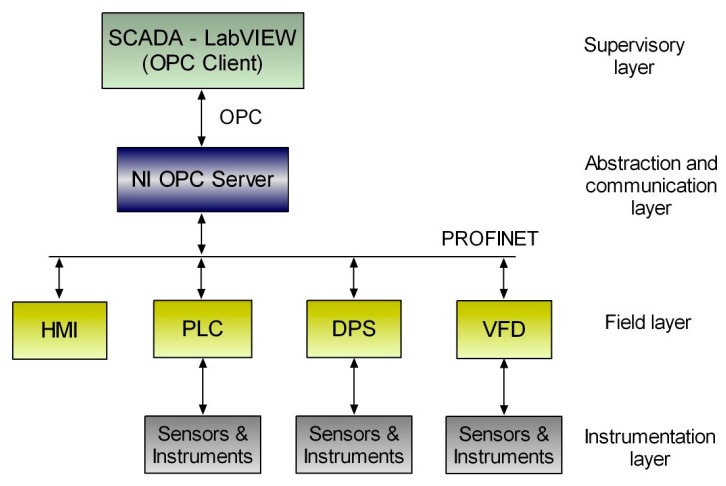
OPC-based communication architecture for photobioreactor automation and supervision.

**Figure 12:**
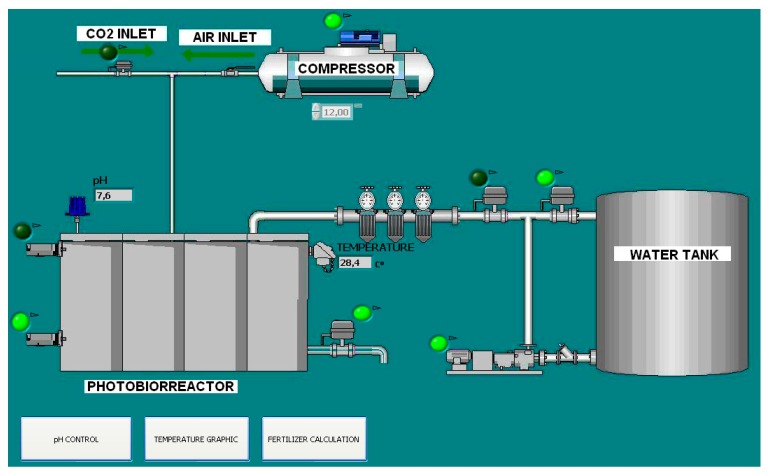
Main screen of the SCADA for the biomass photobioreactor.

The PLC, the DPS, the VFD, and the HMI panel and the DECLS are integrated in a fieldbus PROFINET network. In addition, the computer that runs the OPC server and the SCADA system, DECLS, is included in such network. Figure 11 outlines the communication network according to the proposed architecture. Figure 12 shows the main screen of the SCADA.

### 4.3. Implementation of a NRL

**Figure 13:**
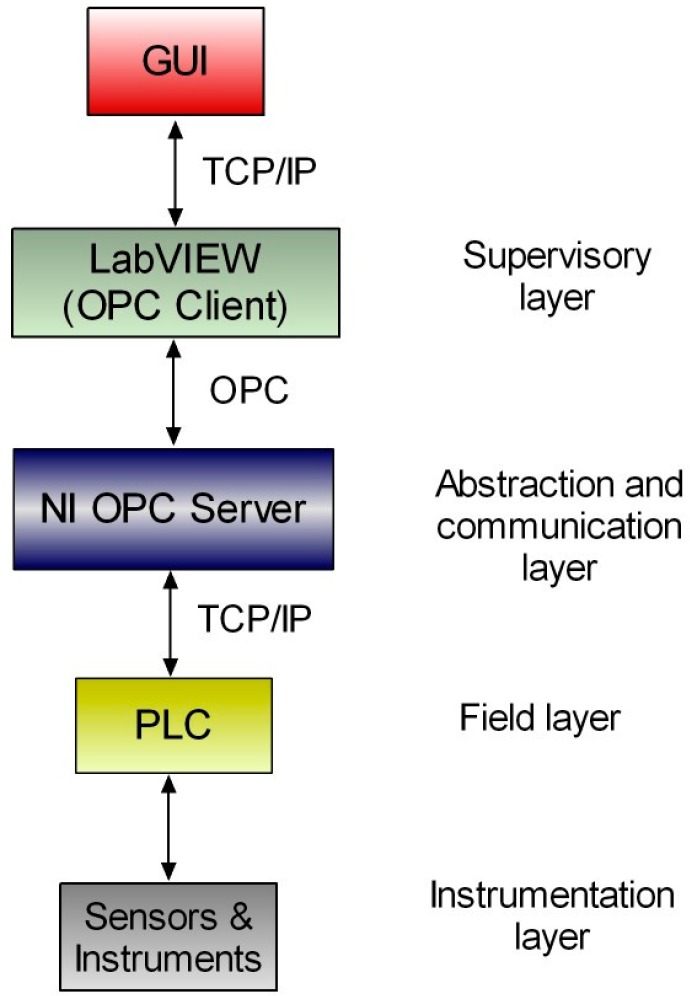
OPC-based communication network for the NRL.

For a proper usage of the developed NRL, the remote user requires not only to observe the plant behavior but also to access the PLC memory in order to parameterize the controller and the experiment in real-time. To solve these requirements, an OPC-based communication network has been developed according to the proposed architecture (Figure 13). 

An OPC linkage is performed between the VI and the PLC. To this aim, the OPC server accesses the signals involved in the control of the DCSM and makes them available for the OPC client. Such role is fulfilled by this VI, so it handles the transmission of information from the OPC server to the GUI. Table 5 displays the magnitudes involved in the remote laboratory and the corresponding devices.

**Figure 14:**
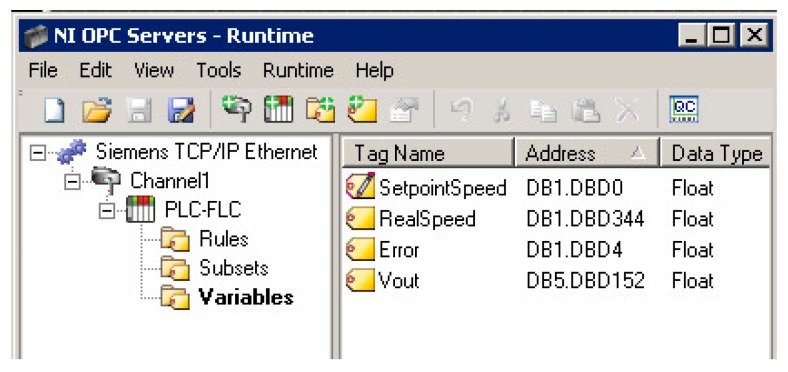
OPC server configuration for data sharing between FLBC/PLC and VI.

**Figure 15:**
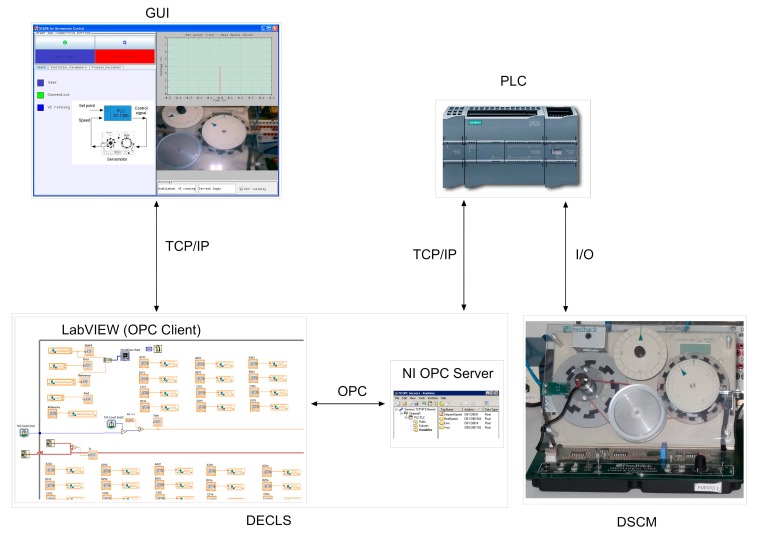
Diagram of the architecture of the developed NRL

Figure 14 shows a sample screenshot of the OPC server configuration. As can be observed, according to the FLBC structure, three groups of tags have been introduced, for the fuzzy rules, for the fuzzy subsets and for the Input/Output (I/O) signals of the controller. Figure 15 illustrates the described scheme. 

### 4.4. HIL Platform with Educational Purposes

**Figure 16:**
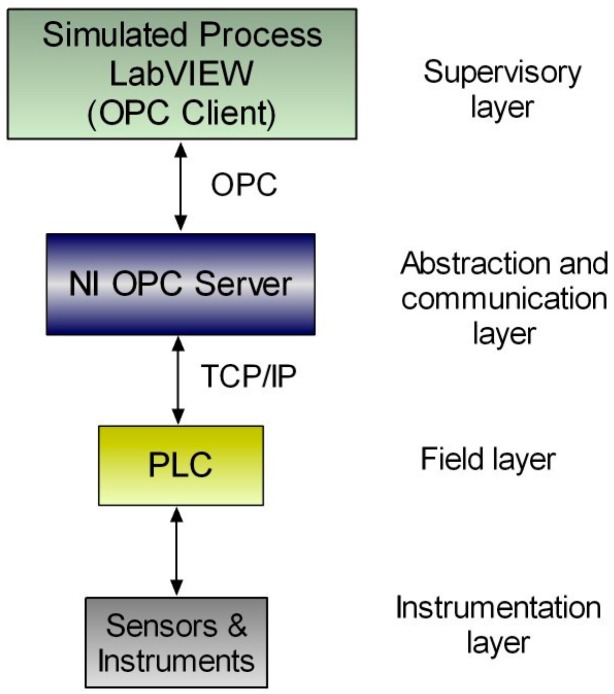
Communication network of the HIL platform.

In the HIL platform, the communication between the PLC and the simulated plant in LabVIEW is performed by the OPC protocol. Figure 16 shows the block diagram of the communication network for the developed platform according to the proposed architecture. As can be seen, the communication between the software simulation environment and the real device is carried out by an OPC linkage. Physically, the PLC and the PC are connected via Ethernet. 

The OPC server makes the PLC variables available for the OPC client, namely a LabVIEW VI. The LabVIEW-based simulation implements a tank with level sensors, electro valves and pumps. The PLC executes a program to control the tank level commanding the valves and pumps from the information provided by the sensors. The own PLC starts the filling of the tank. 

**Figure 17:**
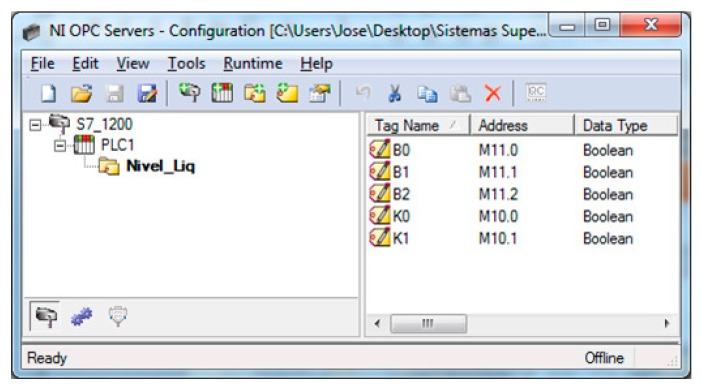
OPC server configuration for data exchange between PLC and simulated process.

The PLC code is devoted to control the process whereas the VI is responsible of animating the simulation. In addition, this VI acts as supervisory system so the student receives real-time information about the plant evolution. The variables exchanged for control tasks are the I/O signals of the plant, namely the level sensors and the commands for electro valves. On the other hand, the simulation requires sharing the tank level and the pipes flow. The I/O signals of the PLC are no connected to sensors neither actuators. Instead of that, these signals are exchanged with the simulated process using an OPC link. As a consequence, in this case, the instrumentation layer of the architecture is virtualized. Figure 17 shows the configuration of the OPC server to establish the information flow between the real controller and the simulated process. Table 6 displays the magnitudes involved in the OPC connection between the virtual plant and the real controller.

**Figure 18:**
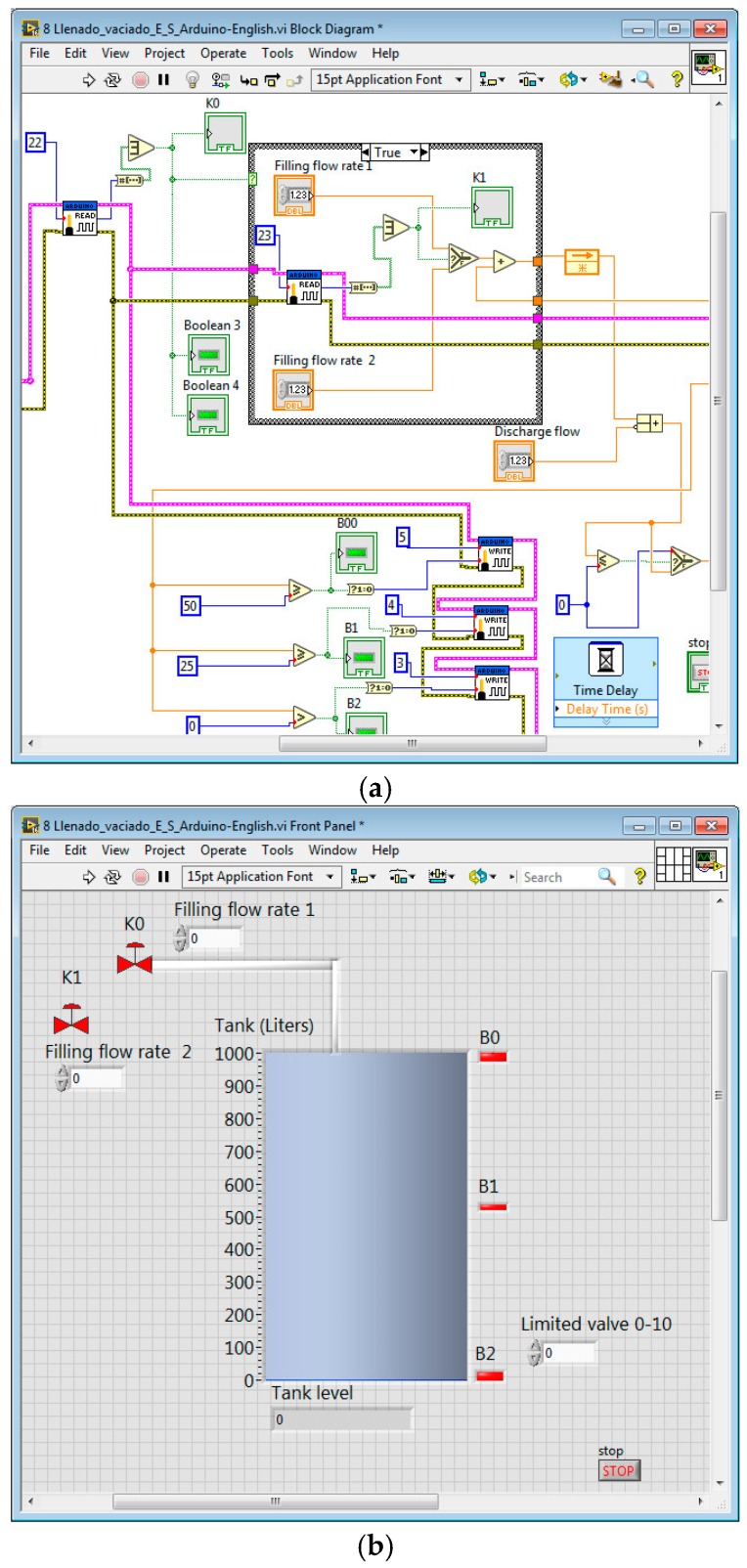
VI provided to students to develop the plant simulation within the HIL platform: (**a**) Graphical code; (**b**) Front panel.

This platform has been successfully applied during the academic course 2014/2015 in two different courses, Supervisory Systems, and Supervisory Control Systems of the Engineering degrees in the UEX. It has been used for the training sessions of both courses. Students have to design and check a PLC program to automate the filling process of a liquid tank. The VI includes several inputs and outputs such as electro valves, level sensors, pumps and so on. The teacher provides a VI with the basic configuration required so students have to complete and improve it. Figure 18 shows the graphical code (Figure 18a) and the front panel (Figure 18b) of such incomplete VI. The HIL platform acts as easy-to-use test bench for the students’ designs. 

**Figure 19:**
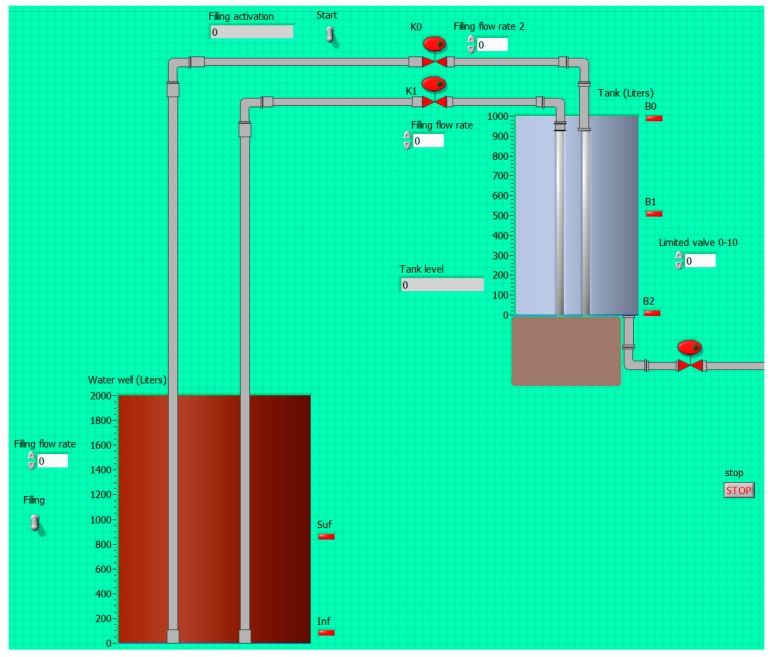
Front panel of VI developed by a sample group of students.

Figure 19 contains the front panel of the VI developed as solution by a sample group of students. This application demonstrates that the OPC interface acts as fundamental tool in the education of future engineers in supervisory systems, industrial networks and systems integration. 

Table 7 summarizes the correspondence between the layers of the proposed architecture and the elements that implement them in the exposed experimentation cases. The instrumentation layer has not been included due to the numerous devices that are already listed in the previous tables.

## 5. Conclusions

The protocol OPC plays an essential role in the field of measurement, monitoring and automation, enabling systems integration for both hardware and software levels. The management of interoperability is a crucial issue both for legacy facilities and for modern and future systems (IoT, CPS, SGs, etc.) that rely on effective data acquisition and transmission. In this context, OPC acts as a tool to facilitate the data flow between the different subsystems. In other words, the data are translated into a common language so every hardware/software element can communicate with the others.

This paper has presented a novel OPC-based architecture to implement automation systems integrating sensors, instruments and controllers. The novelty of the proposal relies in the categorization of the different elements into four functional layers, so it is a new conceptual framework to promote the systematic design and implementation of automation systems involving OPC communication. In addition, the most recent successful application cases developed at the UEX have been reported. In this sense, the automation of energy systems like a smart microgrid and photobioreactor facilities, the implementation of a network-accessible industrial laboratory and the development of an educational hardware-in-the-loop platform have been reported. These cases cover a variety of domains (energy automation, remote laboratories, and education) and involve different hardware and software elements, demonstrating the ability of the proposed architecture to support interoperability.

Thanks to the diverse advantages provided by the OPC interface, the proposed architecture offers features like open connectivity, reliability, scalability, and flexibility.

An important characteristic of the reported experimental applications, despite the fact of being at laboratory scale, is that they use real industrial devices for automation, sensing and data acquisition. For instance, the used PLCs can be found in numerous industrial plants worldwide. In the same sense, all the signals and magnitudes managed are also real, except, obviously, in the case of the HIL platform. Regarding the software packages, both Siemens WinCC flexible and NI LabVIEW are widely applied in the industrial domain for monitoring and supervision tasks.

The paper aims to contribute to foster the deployment of OPC-based systems, acting as a useful resource both for practitioners and researchers in the design of the sensing and automation infrastructures needed to develop their activities. 

Recent trends in R&D activities about OPC are focused on security improvements [87], integration with other protocols like Unified Modeling Language (UML) [45,88], IEC 61850 [89] or Automation Markup Language (AutomationML) [90], and specifications for hard real-time applications [91].

Future guidelines include the utilization of the UA specification for managing a Flexible Manufacturing System under the CPPS context. The integration of IoT-based sensors within the proposed architecture is also a future work to perform. Moreover, the application of the presented architecture in large-scale industrial real scenarios will be an interesting issue to improve the proposal. 

## Figures and Tables

**Table 1:** Summary of the experimental OPC-based systems developed in the UEX.

System	Scope	OPC Role	OPC Server	OPC Client	Hardware
Smart Microgrid	Energy automation	Data exchange for control	WinCC flexible	Matlab/Simulink	Siemens PLC S7-300, Windows-based PC
Biomass Photobioreactor	Energy automation	Monitoring & Control	NI OPC Servers	LabVIEW with DSC module	Siemens PLC S7-1200, Windows-based PC
Networked Remote Laboratory	Industrial applications	Monitoring & Control	NI OPC Servers	LabVIEW with DSC module	Siemens PLC S7-1200, Windows-based PC
Hardware in-the-Loop Platform	Education	Data exchange for control	NI OPC Servers	LabVIEW with DSC module	Siemens PLC S7-1200, Windows-based PC

**Table 2:** List of the magnitudes and devices in the microgrid.

Magnitude	Device
Voltage	DC and AC Converters
Current	Current Transducer
Temperature	Pt100 probe
Pressure	Pressure meter
Hydrogen flow	Flow meter
Wind speed	Anemometer
Solar irradiance	Piranometer
State of Charge	Numerical algorithm

**Table 3:** List of the magnitudes of the microgrid communicated through OPC.

Magnitude
PVGS Current
PEMEL Current
PVGS Temperature
H_2_ Pressure
Solar irradiance
State of Charge
Output of FLBC

**Table 4:** Magnitudes and devices in the photobioreactor communicated through OPC.

Magnitude	Device
CO_2_	Electro valves
Temperature	Pt100
pH	pHmeter
Water level	Electro valves & Capacitive Sensor

**Table 5:** Magnitudes and devices in the NRL communicated through OPC.

Magnitude	Device
DCSM Speed	Tachometer
Setpoint	GUI
Error	Numerical calculation
FLBC parameters	GUI
FLBC Output	PLC

**Table 6:** Magnitudes exchanged via OPC in the HIL platform.

Magnitude
Level sensor 1
Level sensor 2
Level sensor 3
Valve 1
Valve 2

**Table 7:** Summary of layers of the proposed architecture and the experimentation cases.

Layer	Microgrid	Photobioreactor	NRL	HIL Platform
Supervisory layer	FLBC (Matlab/Simulink)	SCADA (LabVIEW)	SCADA (LabVIEW)	SCADA (LabVIEW)
Abstraction and communication layer	WinCC flexible and PROFIBUS	NI OPC Servers and PROFINET	NI OPC Servers	NI OPC Servers
Field layer	PLC S7-300 and DPS	PLC S7-1200 and DPS	PLC S7-1200	PLC S7-1200

## Abbreviations

The following abbreviations are used in this manuscript:

6LoWPAN	IPv6 over Low power Wireless Personal Area Networks
AS-i	Actuator Sensor Interface
AutomationML	Automation Markup Language
CAN	Controller Area Network
CoAP	Constrained Application Protocol
CPPS	Cyber-Physical Production System
DAQ	Data Acquisition Card
DECLS	Data Exchange and Configuration Laboratory Server
DNP3	Distributed Network Protocol
DPS	Decentralized Periphery Station
DSC	Datalogging and Supervisory Control
EESS	Electrochemical Energy Storage System
EJS	Easy Java Simulations
EtherCAT	Ethernet for Control Automation
FLBC	Fuzzy Logic-Based Controller
GUI	Graphical User Interface
HESS	Hydrogen Energy Storage System
HART	Highway Addressable Remote Transducer
HIL	Hardware-in-the-Loop
HMI	Human Machine Interface
HTTP	HyperText Transfer Protocol
ICT	Information and Communications Technologies
IES	Industrial Engineering School
IoT	Internet of Things
IP	Internet Protocol
I/O	Input/Output
LabVIEW	Laboratory Virtual Instrumentation Engineering Workbench
LAN	Local Area Network
M2M	Machine to Machine
NI	National Instruments
NFC	Near Field Communication
NRL	Networked Remote Laboratory
OPC	Object Linking and Embedding for Process Control
OPC DA	OPC Data Access
OPC UA	OPC Unified Architecture
PC	Personal Computer
PEMEL	Polymer Electrolyte Membrane Electrolyzer
PEMFC	Polymer Electrolyte Membrane Fuel Cell
PHM	Prognostics and Health Management
PLC	Programmable Logic Controller
PROFIBUS	PROcess FIeld BUS
PROFINET	PROcess FIeld NETwork
PVGS	Photovoltaic Generator System
RFID	Radio Frequency IDentification
RES	Renewable Energy Sources
RL	Remote Laboratory
RTU	Remote Terminal Unit
SCADA	Supervisory Control and Data Acquisition
TCP	Transmission Control Protocol
VFD	variable Frequency Drive
VI	Virtual Instrument
WGS	Wind Generator System
WSAN	Wireless Sensors and Actuators Networks
WSN	Wireless Sensors Networks
